# Supplementary material for: Joint Association of Dietary Protein Intake and Eating Habits with the Risk of Gestational Diabetes Mellitus: A Case-Control Study
Source: Nutrients. 2023 Oct 11;15(20):4332. doi: 10.3390/nu15204332 (PMC10610363; doi:10.3390/nu15204332)
Supplement: Supplementary file 1 [file nutrients-15-04332-s001.zip › nutrients-2613979-supplementary.pdf]

Supplementary Materials

Supplementary Figure S1.

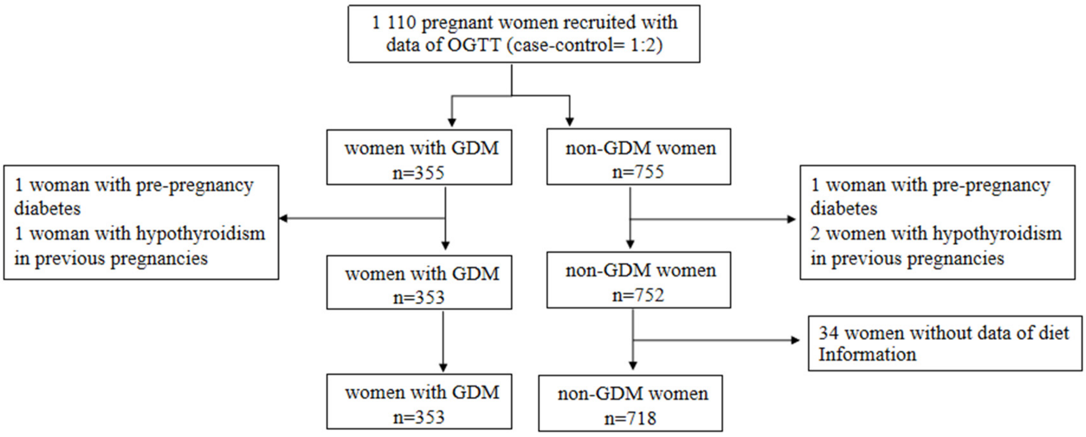

Supplementary Figure S1: Flow chart of participants in our current study.

Supplemental Table S1. Characteristics of participants in our current study [n(%)].

Variables	Total (*n*=1071)	GDM (*n*＝353)	Control (*n*＝718)	*P* value[a]
Age (years)	30.90 ± 3.93	31.97 ± 3.71	30.37 ± 3.94	＜0.001
Pre-pregnancy BMI	22.42 ± 3.82	23.36 ± 3.87	21.96 ± 3.71	＜0.001
Ethnicity				0.904
Han	1052 (98.2)	347 (98.3)	705 (98.2)	
Other	19 (1.8)	6 (1.7)	13 (1.8)	
Marital Status				0.230
Married	1059 (98.9)	351 (99.4)	708 (98.6)	
Unmarried or other	12 (1.1)	2 (0.6)	10 (1.4)	
Annual household income (yuan)				0.385
< 50,000	159 (14.9)	61 (17.3)	98 (13.7)	
50,000～99,900	369 (34.5)	114 (32.3)	255 (35.6)	
100,000～199,900	387 (36.2)	123 (34.8)	264 (36.8)	
⩾ 200,000	155 (14.5)	55 (15.6)	100 (13.9)	
Education status				0.766
Middle school and below	176 (16.4)	63 (17.8)	113 (15.7)	
high school or technical secondary college	223 (20.8)	72 (20.4)	151 (21.0)	
Junior college or regular college	672 (62.7)	218 (61.8)	454 (63.2)	
Working Status				0.693
Mental labor	585 (54.3)	194 (55.0)	391 (54.4)	
Manual labor	69 (6.6)	19 (5.4)	50 (7.0)	
Currently not working	417 (39.1)	140 (39.7)	277 (38.6)	
Smoking				0.525
Yes	1031 (96.3)	338 (95.8)	693 (96.5)	
No	40 (3.7)	15 (4.2)	25 (3.5)	
Drinking				0.327
Yes	899 (83.9)	302 (85.6)	597 (83.1)	
No	172 (16.1)	51 (14.4)	121 (16.9)	
Age of menarche (years)				0.090
8～11	33 (3.1)	18 (5.1)	15 (2.1)	
12	207 (19.3)	65 (18.4)	142 (19.8)	
13	289 (27.0)	90 (25.5)	199 (27.7)	
14	257 (24.0)	88 (24.9)	169 (23.5)	
⩾ 15	285 (26.6)	92 (26.1)	193 (26.9)	
Conception Season				0.138

Spring	287 (26.8)	106 (30.0)	181 (25.2)	
Autumn	166 (15.5)	43 (12.2)	123 (17.1)	
Summer	154 (14.4)	52 (14.7)	102 (14.2)	
Winter	464 (43.3)	152 (43.1)	312 (43.5)	
Adverse maternal history				0.449
No	922 (86.1)	300 (85.0)	622 (86.6)	
Yes	149 (13.9)	53 (15.0)	96 (13.4)	
Family history of diabetes				0.001
No	792 (73.9)	239 (67.7)	533 (77.0)	
Yes	51 (4.8)	99 (28.0)	129 (18.0)	
Unclear	228 (21.3)	15 (4.2)	36 (5.0)	
Family history of cardiovascular				0.089
No	833 (77.8)	278 (78.8)	555 (77.3)	
Yes	175 (16.3)	62 (17.6)	113 (15.7)	
Unclear	63 (5.9)	13 (3.7)	50 (7.0)	
Physical activity				0.229
Low	298 (27.8)	203 (28.3)	95 (26.9)	
Medium	562 (52.5)	365 (50.8)	197 (55.8)	
High	211 (19.7)	150 (20.9)	61 (17.3)	
Sleep quality				0.024
Excellent	923 (86.2)	632 (88.0)	291 (82.4)	
Good	129 (12.0)	77 (10.7)	52 (14.7)	
Average	19 (1.8)	9 (1.3)	10 (2.8)	

Supplemental Table S2. Nutrients intakes according to quartiles of total protein intake in 1,071 participants[a]（$\overline{X} \pm S$）.

Nutrients	Total (*n*=1071)	Protein Intake Quartiles				*P*-value[b]
		Q1	Q2	Q3	Q4	
Dietary intake						
Grain（g/d)	249.30 ± 115.66	135.17 ± 36.80	213.41 ± 24.34	265.78 ± 25.01	384.52 ± 136.89	＜0.001
Beans（g/d)	18.33 ± 24.45	1.63 ± 2.01	7.59 ± 1.11	16.64 ± 3.34	51.05 ± 33.42	＜0.001
Nuts（g/d)	11.87 ± 12.92	0.00 ± 0.00	5.19 ± 2.72	14.55 ± 3.21	30.55 ± 9.66	＜0.001
Soy milk（g/d)	76.64 ± 89.37	0.00 ± 0.00	34.54 ± 16.65	92.49 ± 14.59	213.69 ± 77.52	＜0.001
Red meat（g/d)	59.14 ± 56.64	8.37 ± 4.77	27.74 ± 4.14	50.24 ± 8.32	135.89 ± 52.35	＜0.001
Poultry（g/d)	26.03 ± 39.47	3.37 ± 2.75	11.88 ± 2.42	23.67 ± 4.58	79.68 ± 60.03	＜0.001
Eggs（g/d)[c]	49.18 ± 29.28	46.82 ± 30.41		52.68 ± 27.18		＜0.001
Milk and milkproducts（g/d)	254.11 ± 155.25	81.23 ± 66.91	241.52 ± 17.85	291.23 ± 16.48	459.17 ± 123.77	0.005
Fish（g/d)	28.61 ± 37.50	2.57 ± 2.09	11.84 ± 2.88	24.54 ± 5.77	77.02 ± 48.24	＜0.001
Shrimp（g/d)	23.35 ± 27.48	3.11 ± 2.80	12.51 ± 2.18	24.43 ± 4.39	61.09 ± 35.90	＜0.001
Others nutrient intake						
Total calories(kcal/d)	1546.68 ± 495.75	1118.01 ± 244.12	1408.67 ± 228.25	1627.14 ± 304.48	2027.24 ± 582.61	＜0.001
Carbohydrate(g/d)	255.31 ± 95.04	198.69 ± 55.06	240.49 ± 58.21	266.75 ± 74.67	314.56 ± 130.77	＜0.001
Total fat(g/d)	31.95 ± 13.28	19.67 ± 5.65	27.05 ± 5.43	34.04 ± 5.94	46.85 ± 14.39	＜0.001
Cholesterol(mg/d)	480.16 ± 215.16	309.04 ± 133.72	430.59 ± 141.36	522.37 ± 159.45	658.79 ± 238.30	＜0.001
Total protein(g/d)	71.13 ± 26.66	43.09 ± 8.06	59.95 ± 3.92	74.62 ± 4.94	106.95 ± 23.13	＜0.001
Plant protein(g/d)	30.65 ± 14.02	17.08 ± 3.50	25.03 ± 186	31.63 ± 2.17	48.91 ± 14.78	＜0.001
Animal protein(g/d)	40.48 ± 20.29	20.05 ± 5.31	31.69 ± 2.73	42.07 ± 3.69	68.18 ± 18.33	＜0.001
Protein sources						

From grain(g/d)	21.43 ± 9.87	11.66 ± 2.98	17.99 ± 1.41	22.67 ± 1.54	33.57 ± 11.18	＜0.001
From beans(g/d)	6.41 ± 8.56	0.57 ± 0.70	2.66 ± 0.39	5.83 ± 1.17	17.87 ± 11.70	＜0.001
From nuts(g/d)	1.42 ± 1.55	0.00 ± 0.00	0.62 ± 0.33	1.75 ± 0.38	3.67 ± 1.16	＜0.001
From soy milk(g/d)	1.38 ± 1.61	0.00 ± 0.00	0.62 ± 0.30	1.66 ± 0.26	3.85 ± 1.40	＜0.001
From red meat(g/d)	12.01 ± 11.50	1.70 ± 0.97	5.63 ± 0.84	10.20 ± 1.69	27.59 ± 10.63	＜0.001
From poultry(g/d)	5.21 ± 7.89	0.67 ± 0.55	2.38 ± 0.48	4.73 ± 0.92	15.94 ± 12.01	＜0.001
From eggs(g/d)[c]	6.54 ± 3.89	5.31 ± 2.19		13.45 ± 4.12		＜0.001
From dairy and dairy products(g/d)	7.40 ± 4.55	2.27 ± 1.88	7.18 ± 0.60	8.51 ± 0.41	13.31 ± 3.71	＜0.001
From fish(g/d)	5.08 ± 6.65	0.46 ± 0.37	2.10 ± 0.51	4.36 ± 1.02	13.65 ± 8.55	＜0.001
From shrimp(g/d)	4.25 ± 5.00	0.57 ± 0.51	2.28 ± 0.40	4.45 ± 0.80	11.12 ± 6.53	＜0.001

[a] Values were presented as mean ± SD.

[b] P-values are from ANOVA.

[C] Eggs were classified into two groups.

Supplemental Table S3.:Distribution of dietary habits in different groups [n(%)].

Group	Total （*n*=1071）	GDM （*n*＝353）	Control （*n*＝718）	χ2/t value	*P* value
Food temperature				14.454	0.001
Hot	110（10.3）	54（15.3）	56（7.8）		
Moderate	876（81.8）	272（77.1）	604（84.1）		
Cold	85（7.9）	27（7.6）	58（8.1）		
Firmness				15.535	＜0.001
Firm	159（14.8）	67（19.0）	92（12.8）		
Moderate	720（67.2）	209（59.2）	511（71.2）		
Soft	192（17.9）	77（21.8）	115（16.0）		
Eating speed				7.360	0.025
Fast	224（20.9）	90（25.5）	134（18.7）		
Moderate	718（67.0）	227（64.3）	491（68.4）		
Slow	129（12.0）	36（10.2）	93（13.0）		

Supplemental Table S4:Intake of dairy protein from different source in different group（$\overline{X} \pm S$）.

**Nutrients**	**Total (*n*=1071)**	**GDM (*n*＝353)**	**Control (*n*＝718)**	***P* value[a]**
Dairy Protein Source, g/day				
From milk	6.33 ± 4.24	6.99 ± 4.28	6.01 ± 4.18	＜0.001
From yogurt	1.07 ± 1.45	0.99 ± 1.40	1.11 ± 1.48	0.207

[a] P-values are from ANOVA.
